# Radiosensitivity of Breast Cancer Cells Is Dependent on the Organ Microenvironment

**DOI:** 10.3389/fonc.2022.833894

**Published:** 2022-05-12

**Authors:** Genyan Guo, Ryan T. Morse, Jie Wang, Xuan Chen, Jiajie Zhang, Andrew Z. Wang

**Affiliations:** ^1^ Laboratory of Nano- and Translational Medicine, Lineberger Comprehensive Cancer Center, Carolina Center for Cancer Nanotechnology Excellence, Carolina Institute of Nanomedicine, University of North Carolina at Chapel Hill, Chapel Hill, NC, United States; ^2^ Department of Radiation Oncology, The Fourth Affiliated Hospital of China Medical University, Shenyang, China; ^3^ Department of Radiation Oncology, University of North Carolina Chapel Hill, Chapel Hill, NC, United States; ^4^ Department of Radiation Oncology, Dalian Municipal Central Hospital, Dalian, China; ^5^ Department of Radiation Oncology, Qilu Hospital of Shandong University, Jinan, China; ^6^ Department of Pancreatic Surgery, Huashan Hospital, Fudan University, Shanghai, China

**Keywords:** engineer metastases, breast cancer, radiation response, tumor microenvironment, biomatrix scaffolds, decellularized

## Abstract

**Background:**

Distant metastasis is the leading risk factor of death in breast cancer patients, with lung and liver being commonly involved sites of distant seeding. Ongoing clinical trials are studying the benefit from additional local treatment to these metastatic sites with radiation therapy. However, little is known about the tissue-specific microenvironment and the modulating response to treatments due to limitations of traditional *in vitro* systems. By using biomatrix scaffolds (BMSs) to recreate the complex composition of extracellular matrices in normal organs, we chose to study the radiotherapy response with engineered breast cancer “metastases” in liver and lung organ-specific tissues.

**Methods:**

Liver and lung BMSs were prepared for tissue culture. Human breast cancer cell lines were passaged on normal tissue culture plates or tissue culture plates coated with Matrigel, liver BMSs, and lung BMSs. Clonogenic assays were performed to measure cell survival with varying doses of radiation. Reactive Oxygen Species (ROS) detection assay was used to measure ROS levels after 6 Gy irradiation to cancer cells.

**Results:**

The response of breast cell lines to varying doses of radiotherapy is affected by their *in vitro* acellular microenvironment. Breast cancer cells grown in liver BMSs were more radiosensitive than when grown in lung BMSs. ROS levels for breast cancer cells cultured in lung and liver BMSs were higher than that in plastic or in Matrigel plate cells, before and after radiotherapy, highlighting the interaction with surrounding tissue-specific growth factors and cytokines. ROSs in both lung and liver BMSs were significantly increased after radiotherapy delivery, suggesting these sites create prime environments for radiation-induced cell death.

**Conclusions:**

The therapeutic response of breast cancer metastases is dependent on the organ-specific microenvironment. The interaction between tissue microenvironment in these organs may identify sensitivity of therapeutic drug targets and radiation delivery for future studies.

## Introduction

A majority of breast cancers in the United States are confined to the breast at diagnosis while only 6% of breast cancers are metastatic (1). Despite this, distant metastasis is the leading risk factor of death in breast cancer patients, with lung and liver being commonly involved sites of distant seeding (2). In recent years, pursuits have been made to identify patients that can gain benefit from additional local treatment beyond only systemic therapy with metastatic disease. These paradigms have been defined as oligometastatic and oligoprogressive disease (3). Aggressive treatment to these sites may provide cure in those with limited metastatic burden or could limit further progression in patients with largely controlled systemic disease. Ongoing studies are investigating the use of treating these metastatic sites with ablative doses of radiation, defined as stereotactic body radiotherapy (SBRT) (4).

Tissue-specific microenvironments play an important role in modulating the behavior of metastases and can affect the radiotherapy response of tumors (5, 6). The biology underlying these interactions remains poorly understood due to the lack of sufficient experimental models to study organ-specific metastasis (7). The traditional *in vitro* systems are not suitable for the study of tumor metastasis, because the composition of these substrata are highly dissimilar from the tissue-specific microenvironments encountered by metastases (8).

Recent work in tissue engineering has shown that decellularization methods can be utilized to recreate the complex composition of extracellular matrices found in normal organs. This technique with decellularized tissues has been termed “biomatrix scaffolds (BMSs)” and retains >98% of the tissue’s decellularized matrix components and preserves physiological levels of matrix-bound growth factors and cytokines (9). This technique has been used to engineer cancer “metastases” with colorectal cancer cells *in vitro* that closely resemble *in vivo* metastases histologically, molecularly, and phenotypically (10). This work has led to further understanding of the interplay between tissue microenvironment and radiotherapy response with colorectal metastases, however limited data is available with other metastatic primary tumor histologies. Therefore, in this study we chose to study the radiotherapy response with engineered metastases in liver and lung organ-specific tissues from breast cancer cells.

## Materials and Methods

### Perfusion-Based Decellularization of Liver and Lung

Liver and lung BMSs were produced using Sprague-Dawley rats (male, 250–300 g). The rats were anesthetized and given muscle relaxants before the procedure. BMSs were prepared by cannulating the portal vein (liver BMSs) or inferior vena cava (lung BMSs) for perfusion of decellularization reagents. We performed the procedures until fingertip pain irritation would disappear. For decellularization of the lungs, we exposed the chest by cutting the ribs and rolling up the sternum, cannulating the inferior vena cava, ligating the superior vena cava, and cutting the cervical blood vessels. For decellularization of the liver, we exposed the abdomen by making an incision from the pelvis to the sternum, then cut and removed the sternum, cannulated the portal vein (liver BMSs), and cut the inferior vena cava. The vasculature was perfused with basal medium (e.g. serum-free EMEM/1640) until blood was eliminated and then with 250 mL of 1% sodium deoxycholate (SDC) containing 36 units/L phospholipase. Next, organs were perfused with 3.5 M Sodium Chloride (NaCl) until the perfusate was negative for proteins as assessed by optical density (OD 280.) Finally, tissues were rinsed with basal medium and snap frozen. Frozen decellularized organs were pulverized into a fine powder using a freezer mill (Spex SamplePrep 6770, Metuchen, NJ). Processed BMS powder was stored at −80°C.

All animal experiments were conducted in accordance with guidelines provided by University of North Carolina at Chapel Hill (UNC) Institutional Animal Care and Use Committee.

### Preparation of BMSs Coated Tissue Culture Plates

To determine protein concentrations of BMS materials, BMSs were dissolved in a solution composed of 4 M guanidine hydrochloric acid (HCl), 50 mM sodium acetate (pH 5.8), and 25 mM Ethylenediaminetetraacetic acid (EDTA) containing proteinase and phosphatase inhibitor cocktails. Nanodrop method was used to determine total protein concentrations. To prepare BMS coated surfaces, BMSs were suspended in ddH_2_O, added to tissue culture plates (untreated), and allowed to dry overnight. Plates were sterilized using 100 Gy of external beam irradiation (Precision X-Ray). BMSs were prepared by using 3 organs of each liver or lung, respectively, for the tissue cultured plates.

### Cell Culture

Human breast cancer cell lines (BT-549 RRID: CVCL_1092, BT-20 RRID: CVCL_0178) were acquired from the Tissue Culture Facility at UNC Lineberger Comprehensive Cancer Center. BT-549 and BT-20 cell lines are primary tumor invasive ductal carcinomas that were isolated from triple-negative breast cancer patients. Cell lines were authenticated using short tandem repeat and were tested for mycoplasma contamination. BT-549 cells were cultured in 1640 (Gibco) supplemented with 10% fetal bovine serum (Gibco) and penicillin/streptomycin (Mediatech). BT-20 cells were cultured in EMEM (Gibco) supplemented with 10% fetal bovine serum (Gibco) and penicillin/streptomycin (Mediatech). Cells were passaged on normal tissue culture plates or tissue culture plates coated with Matrigel, liver BMSs, and lung BMSs (300-350 ug/cm^2^).

### Clonogenic Assays

Plating efficiency (PE) of each cell line was determined. Cells grown on plastic, Matrigel, liver BMSs, and lung BMSs were irradiated with 0, 2, 4, 6, and 8 Gy. Following irradiation, cells were plated into six-well plates at densities ranging from 100 to 10,000. Cells were incubated for 14 days, fixed, and then stained a solution composed of 4% formaldehyde, 80% methanol, and 0.25% crystal violet. Only colonies containing 30 or more cells were counted. We performed two clonogenic assay experiments with two-replicate wells for each group. The surviving fraction (SF) was calculated using the formula: (# of colonies formed)/(# of plated cells) (Plating Efficiency). The SF was plotted against the radiation dose on a log scale. Linear-quadratic formula SF=e^(−αD-βD2) was used to generate survival curves. Proliferation rates of the different cell lines are shown in the Supplementary material (**Supplementary Figure 1** and **Figure 2**).

### Reactive Oxygen Species (ROS) Detection Assay

BT-549 and BT-20 cells were seeded at 2.5 x 10^4^ cells/well in 96-well plates coated with Matrigel, liver BMSs, lung BMSs and uncoated plates. One-day post seeding, cells were treated with 6 Gy radiation. ROS within cells before and after radiation was determined by dichlorofluorescein diacetate (DCF-DA) cellular reactive oxygen species detection assay using the DCFDA -cellular reactive oxygen species detection assay KIT (Abcam). Treatment response for each culture condition was standardized to untreated cultures. We performed three reactive oxygen species detection assay experiments with five replicate wells for each group.

### Statistical Analysis

Data analysis, including linear-quadratic cell survival curves, was performed using GraphPad Prism 6 (GraphPad). In ROS detection assay, multiple comparisons between two groups were performed using analysis of variance (ANOVA) and least significant difference (LSD) *post hoc* tests. A p-value <0.05 was considered to indicate a statistically significant difference.

## Results

### Creation of Organ-Specific Biomatrix Scaffolds

**Figure 1:**
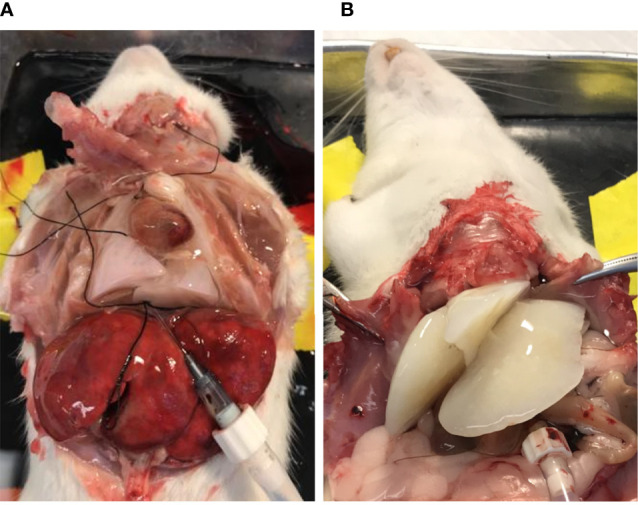
Decellularization of lung **(A)** and liver **(B)** tissues produces BMSs containing tissue specific signaling molecules.

To prepare lung BMSs, we used a perfusion-based extracellular-matrix (ECM) isolation technique (9). The rat’s inferior vena cava (IVC) was cannulated for the infusion of decellularization reagents and the superior vena cava (SVC) was clamped using a vessel clip. An opening was made in the rat’s carotid artery for outflow. The color change of the rat lung (from white to nearly transparent) provided a preliminary indication of successful decellularization (**Figure 1A**). Decellularized liver (**Figure 1B**) BMSs were prepared by cannulating the hepatic portal vein for the infusion of decellularization reagents. Performing this same technique, we decellularized liver tissue to create liver BMSs.

### Breast Cancer Cell Lines Form Liver and Lung “Metastases” *In Vitro*

**Figure 2:**
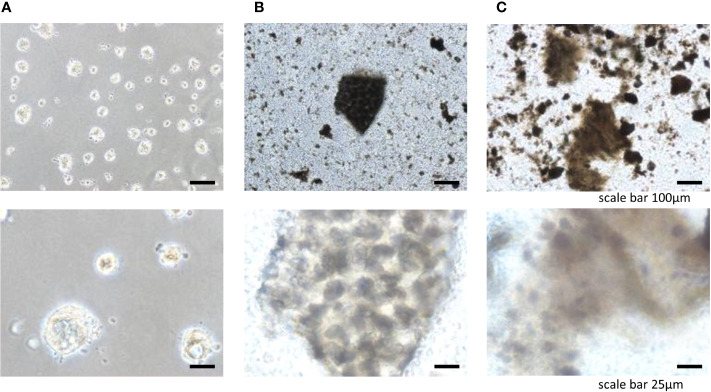
Three-dimensional cell colonies BT20 in **(A)** Matrigel, **(B)** lung, **(C)** liver.

**Figure 3:**
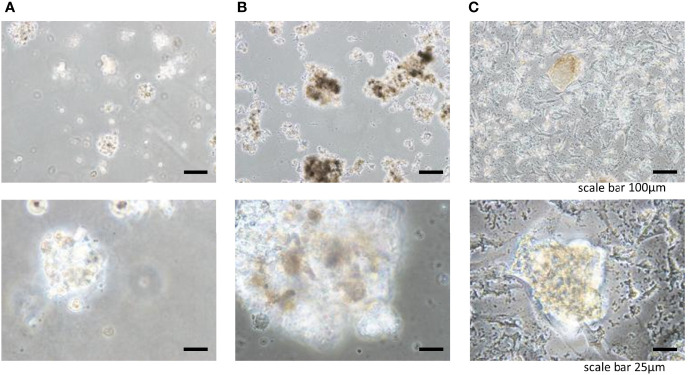
Three-dimensional cell colonies BT549 in **(A)** Matrigel, **(B)** lung, **(C)** liver.

To engineer tissue-specific breast cancer metastases, we cultured breast cancer cell lines (BT-549, BT-20) on tissue culture plates coated with liver and lung BMSs (11). Both breast cancer cell lines (BT-549, BT-20) spontaneously formed 3D spheroid colonies comprised of tumor cells bound together *via* tight junctions (**Figures 2**, **3**). These “metastases” are relatively large in scale, attaining diameters of up to a millimeter. Tumor spheroids that attain a diameter of greater than 500 micrometers contain necrotic cores due to a general lack of oxygen and nutrient availability as well as the internal accumulation of cytotoxic metabolites (12, 13). Consistent with this observation, metastases engineered on our BMSs also contain necrotic regions similar to the hypoxic and necrotic regions found in *in vivo* metastases (10).

### Engineered Metastases Demonstrate Organ-Specific Radiation Response

**Figure 4:**
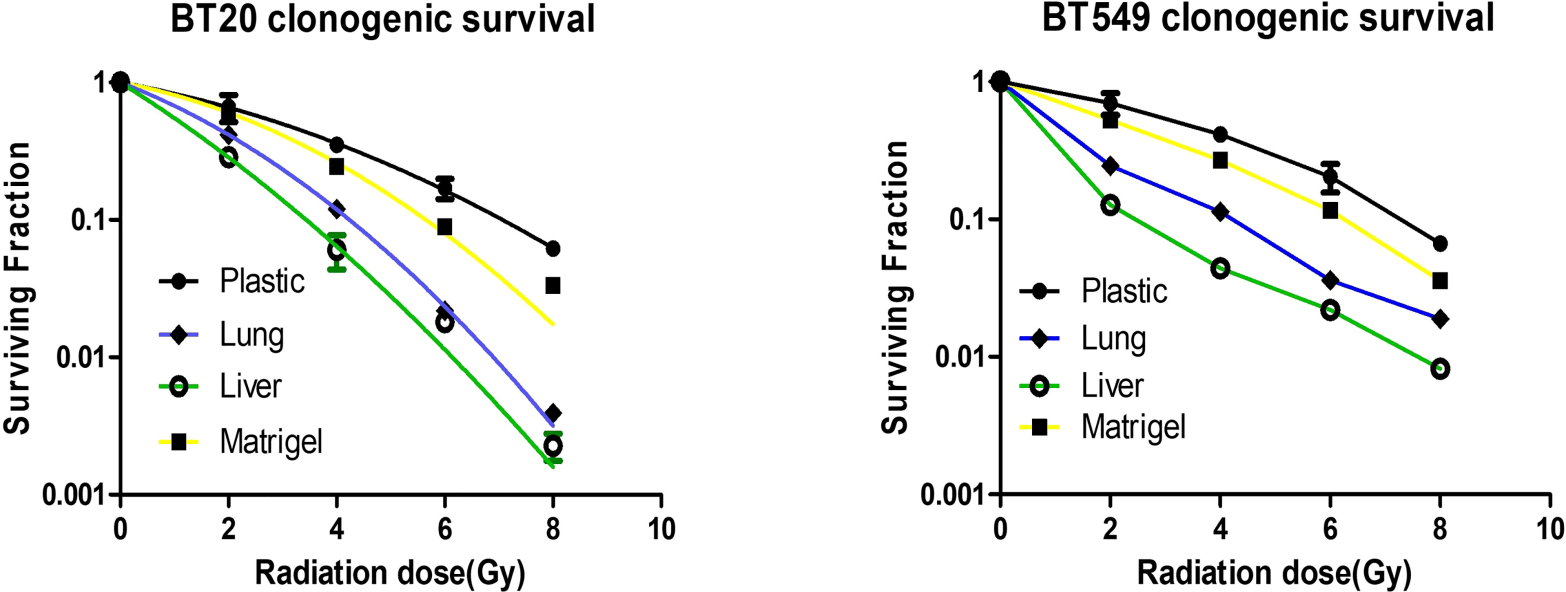
Breast cancer cells grown on different substrata respond differently to radiotherapy. Response of breast cancer cells grown on plastic, Matrigel, liver BMSs, and lung BMSs to radiotherapy (n = 3 biologically independent cell samples). Data represent mean ± S.E.M.

The identification of radiation dose levels that are effective in treating metastases in a tissue-specific manner remains an active area of interest, as metastases in different organs of the same patient can respond differently to varying doses of radiation. To determine if the substrata upon which breast cancer cells are grown affects radiation treatment response, we treated breast cancer cells grown on plastic, Matrigel, liver BMSs, and lung BMSs. We found that the response of breast cell lines to radiotherapy is affected by their *in vitro* acellular microenvironment (**Figure 4**). Importantly, we observed that the treatment response of engineered liver and lung metastases differed. Breast cancer cells grown in liver BMSs were more radiosensitive than when grown in lung BMSs. Our results demonstrate that the radiation response of breast cancer cells is impacted by the organ-specific composition of the BMSs on which they are cultured.

### Engineered Metastases Demonstrate Organ-Specific Levels of ROS

**Figure 5:**
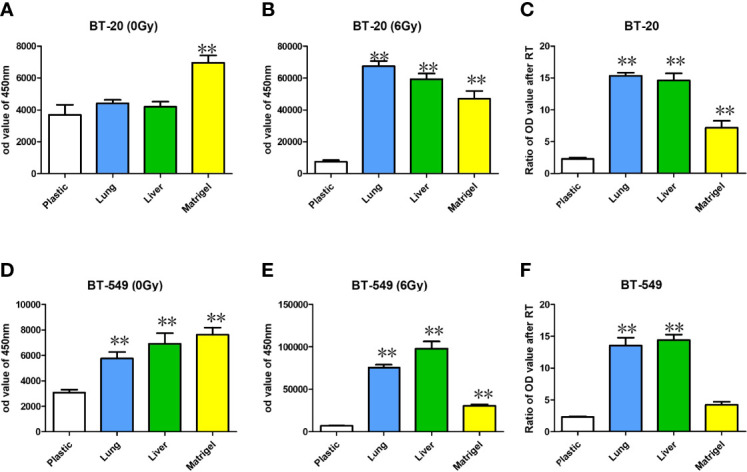
ROS were measured with DCF-DA staining with BT-20 and BT-549 breast cancer cell lines before **(A, D)** and after radiotherapy delivery **(B, E)**. The relative increases in ROS can be found in **(C, F)**. Data represent mean ± S.E.M. * denotes P<0.05, ** denotes P<0.001.

To determine if the substrata upon which breast cancer cells are grown effects ROS, we measured ROS levels in breast cancer cells grown on plastic, Matrigel, liver BMSs, and lung BMSs before and after radiotherapy. We found that the level of ROS in breast cancer cell lines is affected by their *in vitro* acellular microenvironment before(**Figures 5A, D**) and after radiation delivery (**Figures 5B, E**).

ROS in the cells cultured in the BMSs before radiotherapy was higher than that in the cells cultured in the ordinary culture plate. The increase of ROS in the cells cultured in the matrix after radiotherapy was prominent in both lung and liver BMSs. Compared with before and after radiotherapy, the increase of ROS in the cells cultured in the matrix was significantly higher than in the cells cultured in the ordinary culture condition (**Figures 5C, F**).

## Discussion

Despite major breakthroughs in oncologic outcomes in breast cancer, distant recurrence remains a major cause of mortality (2). Oncologists have been pushing the boundary of disease outcomes in metastatic patients with more aggressive treatment, however little is known about treatment response of metastatic cells when they have migrated to other organs than their primary disease site. The tissue-specific microenvironment can strongly influence the local behavior of metastases (14). With this understanding, we used a 3D *in vitro* culture model using decellularized organs to study the interaction of the microenvironment with breast cancer metastases. This work was built off of our previous model using colorectal cancer cells to strongly resemble the *in vivo* behavior of cancer cells (10). Our culture system, capable of engineering metastases *in vitro*, represents a powerful tool to better study metastatic cancer biology in a tissue-specific manner and the effects on treatment response.

The response of metastases to therapeutic treatment, either chemotherapy or radiotherapy, may be influenced by the organ-specific microenvironment (15). The therapeutic response of breast cancer cells in our study was dependent on the tissue specific acellular microenvironment exposure *in vitro*. Our data suggest that breast cancer cells grown in lung and liver BMSs are more sensitive to radiotherapy than cells grown in Matrigel and ordinary culture. We found that engineered metastases on liver BMSs were more sensitive to radiation than when grown on lung BMSs. This finding may suggest that breast cancer cells to the liver may be treated with lower radiation doses than metastases to the lung. Sparing more normal liver tissue would prove vital in patients with metastatic disease typically on chemotherapies metabolized mainly by the liver. This finding is in contrast to our previous results with chemotherapy response in organ-specific BMSs (10). We previously found cancer cells grown on lung BMSs more sensitive to chemotherapy than liver BMSs. This information could aid in treatment decisions for patients when deciding between local radiation therapy or systemic chemotherapy based on location of metastases.

Reactive oxygen species (ROS) are typically upregulated in tumor cells, however tumor cells have evolved mechanisms to maintain proper balance for their survival (16). Radiation works by increasing reactive oxygen species within tumor cells to induce DNA damage, eventually leading to cancer cell death (17, 18). Lower levels of ROS have been correlated with tumor cells that are more radioresistant, such as cancer stem cells (19). In this study, we aimed to detect the level of ROS in tumor cells before and after radiotherapy in different culture conditions. Our results showed that levels of ROS in breast cancer cells cultured in lung and liver BMSs was higher than that in plastic or in Matrigel plate cells, before and after radiotherapy, suggesting the impact on breast cancer cells by their *in vitro* acellular microenvironment. This highlights the interaction with surrounding tissue-specific growth factors and cytokines. Results showed elevated increases in ROSs in both lung and liver BMSs after radiotherapy delivery, suggesting these sites create prime environments for radiation-induced cell death.

The *in vitro* model we have prepared using decellurization methods to create biomatrix scaffolds has limitations. This model cannot fully recapitulate the tumor microenvironment *in vivo*. However, it retains greater than 90% of the matrix components in addition to growth factors and cytokines (9). This culture system capable of engineering metastases represents a powerful tool to further understand the tumor microenvironment in an organ-specific manner. Future study can aim to include multiple cell populations with varying mutational burdens to more accurately represent the biology of metastatic cells. Breast cancer patients often develop bone and brain metastases in addition to lung and liver, identifying the interaction between tissue microenvironment in these organs may identify sensitivity of therapeutic agents, including radiation sensitivity. Identifying specific ECM components that affect cancer cell survival and treatment response will be important to developing new therapeutic drug targets. Our model can be used to screen tissue-specific treatment responses of metastases with newly developed cancer drugs.

## Data Availability Statement

The original contributions presented in the study are included in the article/**Supplementary Material**. Further inquiries can be directed to the corresponding author.

## Ethics Statement

All animal experiments were conducted in accordance with guidelines provided by University of North Carolina at Chapel Hill (UNC) Institutional Animal Care and Use Committee.

## Author Contributions

Conception and design: AW and GG; Administrative support: AW; Provision of study materials or patients: AW; Collection and assembly of data: JW, GG, XC, and JZ; Data analysis and interpretation: GG, RM, JZ, and JW; Manuscript writing: All authors; Final approval of manuscript: All authors.

## Conflict of Interest

The authors declare that the research was conducted in the absence of any commercial or financial relationships that could be construed as a potential conflict of interest.

## Publisher’s Note

All claims expressed in this article are solely those of the authors and do not necessarily represent those of their affiliated organizations, or those of the publisher, the editors and the reviewers. Any product that may be evaluated in this article, or claim that may be made by its manufacturer, is not guaranteed or endorsed by the publisher.

## Supplementary Material

Supplementary Figure 1 BT-549 cancer cells grown on plastic, matrigel, lung BMSs, and liver BMSs at varying levels of radiation dose.

Supplementary Figure 2 BT-20 cancer cells grown on plastic, matrigel, lung BMSs, and liver BMSs at varying levels of radiation dose.
