# Supplementary figures and images for: Radiosensitivity of Breast Cancer Cells Is Dependent on the Organ Microenvironment
Source: Front Oncol. 2022 May 12;12:833894. doi: 10.3389/fonc.2022.833894 (PMC9134193; doi:10.3389/fonc.2022.833894)

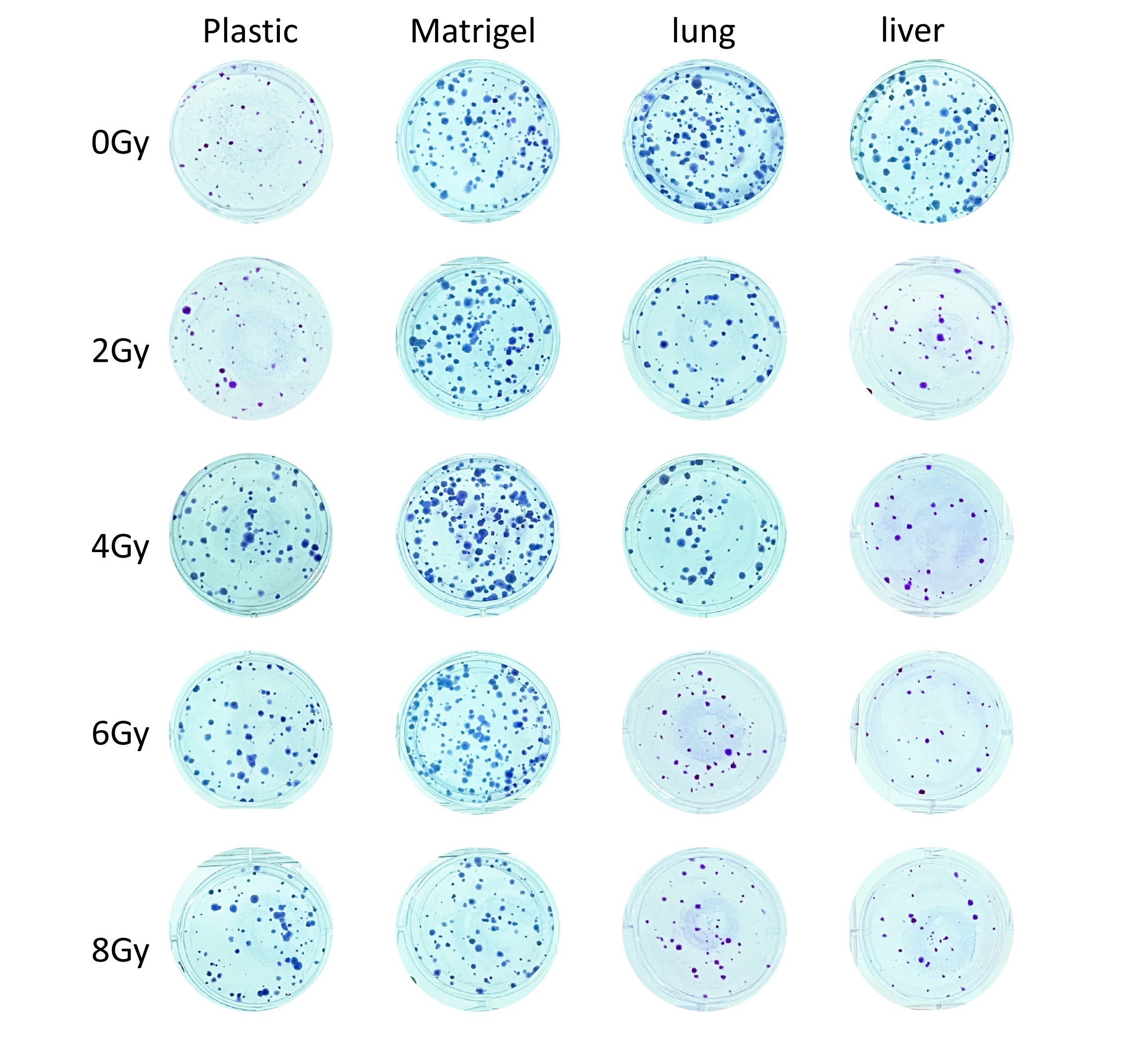

Supplement: Supplementary Figure 1 — BT-549 cancer cells grown on plastic, matrigel, lung BMSs, and liver BMSs at varying levels of radiation dose. [file Image_1.jpeg]

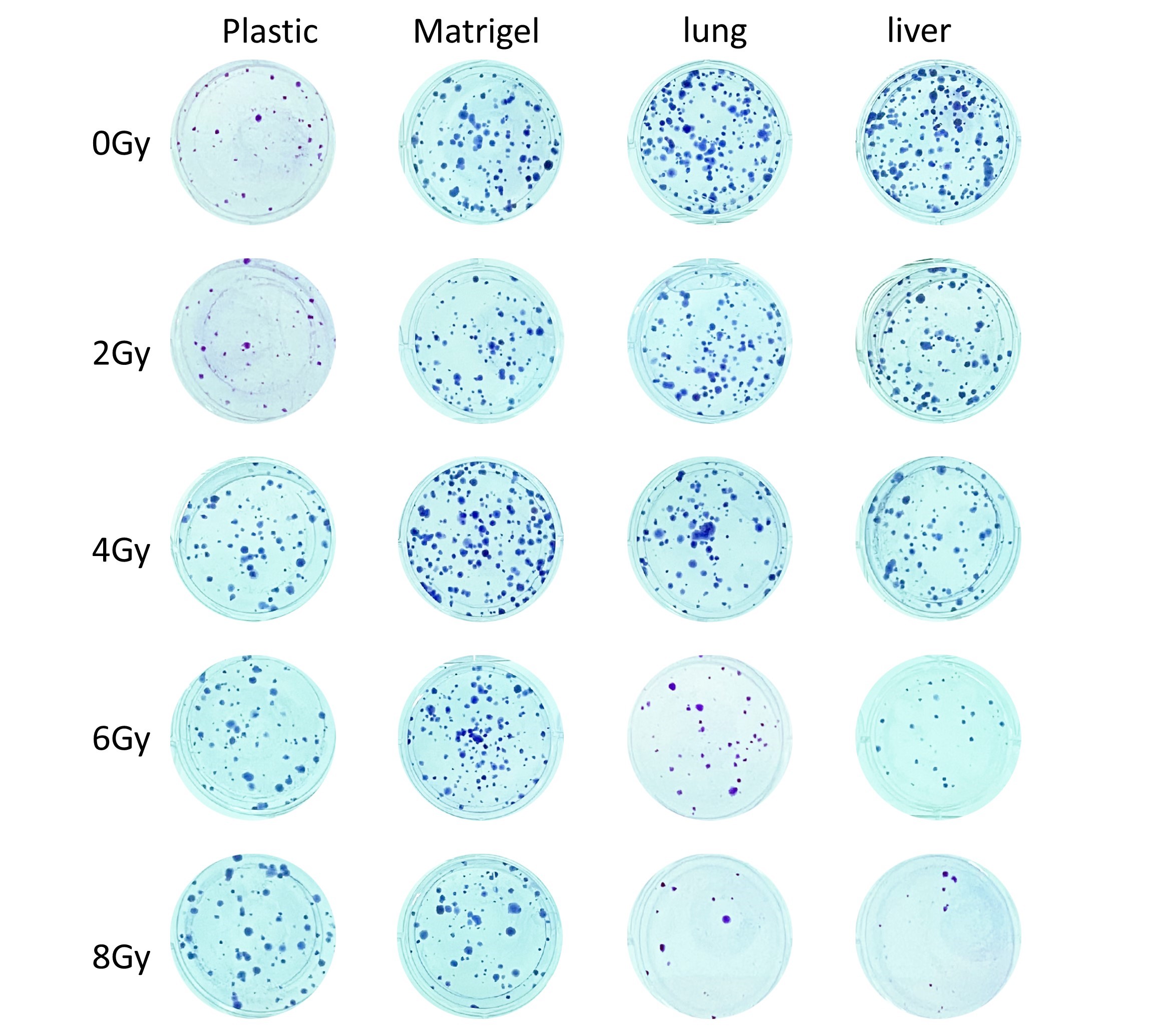

Supplement: Supplementary Figure 2 — BT-20 cancer cells grown on plastic, matrigel, lung BMSs, and liver BMSs at varying levels of radiation dose. [file Image_2.jpeg]
